# Overcoming communication barriers in a multicultural radiography setting

**DOI:** 10.4102/hsag.v26i0.1568

**Published:** 2021-06-04

**Authors:** Cherise Janse van Vuuren, Barbara van Dyk, Padidi L. Mokoena

**Affiliations:** 1 Department of Medical Imaging and Radiation Science, Faculty of Health Sciences, University of Johannesburg, Johannesburg, South Africa

**Keywords:** multicultural, multilingual, diversity, communication barriers, radiographer, healthcare

## Abstract

**Background:**

Effective communication between the patients and radiographers can be a daunting task in a multicultural, multilingual environment. With 11 official languages, South Africans experience language barriers amongst themselves, which pose unique communication challenges on a daily basis. It is thus important to explore how radiographers overcome such challenges to provide an effective service to their patients.

**Aim:**

The aim of this study was to explore and describe the experiences of radiographers in Gauteng province in communicating with patients in a multilingual, multicultural healthcare setting and make recommendations towards overcoming such barriers.

**Setting:**

The focus group discussions were conducted in English and at a private location that was convenient for the participants in Gauteng.

**Method:**

The study employed a qualitative phenomenological approach using focus group interviews (FGIs) to solicit the experiences of participants and gain an in-depth understanding of the phenomenon.

**Results:**

The findings showed that patient–radiographer cross-cultural communication is ineffective whilst language barriers are encountered daily. Participants subsequently offered a number of recommendations to enhance communication with patients from different cultural and linguistic backgrounds. These included workshops or short courses to improve language skills, posters to allow for non-verbal communication, the use of professional interpreters or mobile translation technology, employment of a diverse workforce and a focus on cultural sensitivity and learning an additional language at tertiary level.

**Conclusion:**

Although a variety of communication strategies are available, the most appropriate combination should be explored for individual radiology practices in order to serve their respective diverse patient base. Recommendations that emanated from this study can, therefore, be used as a guide to radiology practices to facilitate effective patient–radiographer communication.

## Introduction

South Africa (SA) is a multicultural and multilingual society with 11 official languages (*Constitution of the Republic of South Africa Act* 1996). In addition, SA is home to a large number of refugees from other African countries, whilst Gauteng is seen as the Mecca of opportunities and jobs for both citizens and foreigners (Census 2011 2012). The Gauteng healthcare setting subsequently embodies a blend of multicultural and multilingual patients and healthcare providers. Whilst English is only the fourth most used language in the country, it is generally the preferred language used in a healthcare setting (Census 2011 2012).

Although the National Language Policy Framework (South African Government 2003) stipulates that each province must meet the specific needs of their population in order to embrace the individual culture of each province, Gauteng, like the rest of South Africa, does not have a single dominant home language (Census 2011 2012). It thus stands to reason that the South African population experiences language barriers even amongst themselves (Claymore 2014), which pose unique communication challenges on a daily basis. In this environment, effective communication between patients and health workers can be a daunting task.

Patients’ perception of the quality of care they receive is highly dependent on the quality of their interactions with their caregivers (Kemp et al. 2017:606). In the radiography department, effective communication between patients and radiographers translates into better clinical histories being obtained, higher quality imaging studies being performed and a reduction in repeat exposures to ionising radiation (Kemp et al. 2017:606). Patient-centred communication subsequently impacts the quality of care and health outcomes, with language and cultural differences being the most debilitating barriers to effective patient-centred medical services (Fernandez et al. 2014:9; Levinson et al. 2010:1320).

Communication is, however, not only limited to a verbal format. Written communication, such as pamphlets, posters and pictures, and non-verbal communication, such as facial expressions and hand gestures, are also used to relay a message between the patient and the healthcare provider (Velentzas & Broni 2014:121).

Trying to achieve this simple aspect of healthcare can, however, be daunting for both patient and provider when a common language is not shared (Meuter et al. 2015:371). As a result, communication challenges between patients and medical staff can result in a depersonalised healthcare experience (Fernandez et al. 2014:9).

The aim of this article is, therefore, to make recommendations towards overcoming communication barriers in a radiography-specific context, based on the results obtained from a phenomenological study.

## Methodology

### Design

A phenomenological qualitative design was utilised in order to explore and describe the experiences of radiographers in Gauteng province when communicating with patients in a multicultural, multilingual setting. This design allowed the complex nature of multicultural communication to be explored and analysed until it was fully understood (Creswell & Poth 2017:24).

### Research setting

This study was conducted in SA through the University of Johannesburg, Department of Medical Imaging and Radiation sciences (MIRS), which resides in the Faculty of Health Sciences. The focus group interviews (FGIs) took place at two separate, neutral and centrally located venues. The two venues were located at two different convenient locations allowing for ease of access for the participants. Venues were selected based on privacy and low noise levels, thus ensuring that no disruptions occurred during the FGI. Each session lasted for between 38 and 50 min.

### Study population and sample

The population (*N* = 7309) consisted of diagnostic radiographers registered with the Health Professions Council of South Africa (HPCSA), working in private or public healthcare settings.

Information-rich subjects were purposefully invited to participate in this study through a snowball sampling technique. A total of 18 radiographers participated in four FGIs by which time data saturation had been reached. The focus groups consisted of 4 to 5 participants each, all of whom were qualified radiographers practising in either the private (*n* = 11) or the public (*n* = 7) sector in Gauteng province, South Africa.

### Data collection

Data collection took place between October and November 2018 at two neutral venues in Gauteng. Each FGI started with the following broad question: ‘Tell me about your experience of having to communicate with patients from different cultures and speaking different languages’. This was then followed by additional probing questions, which emanated from the responses during the FGIs, to explore the participant’s experiences further. The FGIs were conducted in English, recorded on an audio recorder and then transcribed by the researcher.

### Data analysis

Data analysis commenced by listening to the recordings repeatedly until specific words and concepts could be identified. Transcripts were subsequently read into a computer software program ATLAS.ti, which coded the data into broad themes and categories connected to each theme. Field notes, taken during the FGIs, provided additional information about group dynamics and body language. Member checking furthermore ensured that it was clearly understood what was implied by a participant’s response, whilst bracketing allowed the researcher to set aside personal assumptions in order to ensure a pure description of participant’s views (Creswell & Poth 2017:77).

### Measures of trustworthiness

Trustworthiness was ensured through the use of Lincoln and Guba’s (1981) criteria of credibility, dependability, transferability and confirmability. A qualitative study is deemed credible when it provides an accurate description and interpretation of human experiences. Credibility was ensured by using verbatim transcriptions of the FGIs, prolonged engagement with participants, member checking and field notes, which added non-verbalised nuances such as body language and group dynamics. This technique ensured that the data were rich, robust and comprehensive.

To ensure dependability, a detailed account of all aspects of the research process was recorded for future reference. All FGIs were audio-recorded to allow unbiased documentation of verbatim quotes, allowing for limited distortion of data and accurate and detailed descriptions. Transferability was achieved by describing the phenomenon in sufficient detail in order for the reader to evaluate the extent to which the conclusions drawn are transferable to other contexts. Moreover, a detailed account of the strategies used to capture the responses was documented in order to demonstrate confirmability.

### Ethical considerations

Ethical clearance was obtained from the University of Johannesburg Research Ethics Committee (NHREC Registration number: REC-241112-035) and the Higher Degrees Committee of the Faculty of Health Sciences (HDC-01-51-2018). Consent to participate in the study and participant responses to be audio recorded was obtained before commencement of the FGIs.

## Results

Reducing communication barriers should be an important component in the efforts to improve patient care and healthcare service in general (Albrecht et al. 2013:2). It was apparent that the presence of communication barriers between radiographers and patients was an everyday occurrence in the healthcare setting. During the FGIs, participants proposed interventions, specific to a radiology department, which would help overcome communication barriers. These interventions can be categorised into six main themes, which are discussed in the following sections.

### Workshops and short courses

Participants indicated that they were willing to learn a new language in order to help them communicate with patients from different backgrounds. Recommendations ranged from short courses and workshops to informal discussions about language.

There was consensus that new language skills should, however, be specific to the medical field and the radiography setting in particular in order to bridge the communication gap effectively. As there is no need to have a general conversation with the patient, the focus is on obtaining a medical history and on conveying the most important information regarding the procedure. This will enable radiographers to learn what is needed in a shorter time frame whilst learning a completely new language will be time-consuming:

‘It should still be something that is more purpose driven to meet our more specific needs … I’m not saying that learning another language is not a good idea, I’m not knocking that. I’m just saying … you don’t necessarily need to learn another language completely.’ (code 4:1)

‘Ideally we would need someone who is in the medical field or has an understanding of the medical field to teach us terminology that will empower us and not just basics.’ (code 4:5)

‘You need a specifically designed language course with relevancy to our profession.’ (code 4:1)

Interestingly, the level of willingness significantly increased, provided that an incentive was tied to their effort:

‘Interviewer: if you have an incentive, would you be more willing to learn a new language?’

‘Participant: I think so. Yes, the (CPD) points would help.’ (code 3:3)

Participants, nevertheless, recognised the benefit of learning another language towards professional development and as a means to serve their patients better:

‘I would think it would also help with professional development.

Now you’ve got the department learning a language.

It’s more interactive. Everybody gets a skill.’ (code 3:1)

The preference for formal workshops, however, ensued because of the standardisation in teaching it would ensure. Participants held the opinion that it would be easier to learn the correct terms and words during formal training sessions whilst the reluctance between colleagues to take responsibility for teaching a language would be eliminated:

‘It should be standardized throughout the practice. Not someone in our department teaching to say this and someone else says that. It should be standardised.’ (code 4:1)

### Posters and written documents

Posters and visual aids are cost-effective and easy ways to help overcome language barriers. The most common suggestion was a poster indicating the translated phrase or words to help the radiographer communicate with a patient:

‘There was a laminated print out of the basic words just pasted by the exposure panels.’ (code 4:4)

In addition, the use of a guidebook that contains the most used terms translated in a variety of languages was a popular suggestion. The layout should enable quick referencing with all the terms related to a specific examination being grouped together:

‘They could just develop a little guide book and then in that guide book, there are different languages like French, Portuguese, and Chinese. All the major common terms that a radiographer would use in practice.’ (code 3:1)

‘I think a good thing maybe to add to a department is to a make file. Just a small little file with basic language things: “breathe in,” “breathe out.” And then you can always go back to your little file and not just our 11 languages.’ (code 2:1)

Pictures of certain actions, such as coughing or falling, were furthermore suggested in order to help with the history taking process, whilst pictures of body parts were suggested to determine if and where a patient is experiencing pain. In this way, even illiterate patients may benefit because images are self-explanatory:

‘Yes, even pictures or like a poster.’ (code 3:4)

‘…with like areas of the body.’ (code 2:1)

Lastly, flashcards with written instructions were proposed to help communicate with deaf patients. This would allow someone to show the patient the card with instructions, such as ‘inhale’ or ‘swallow’:

‘[*W*]e can always make cards, that says “breathe in” and show it.’ (code 2:2)

### Professional interpreter

The need for professional interpreters was mentioned during all the FGIs. Participants, however, acknowledged that the vast number of languages encountered in Gauteng pose challenges as a single interpreter will not be able to comply with the 11 official languages and foreign languages additionally encountered:

‘If radiology departments employed a translator where, if that area is predominantly Afrikaans or predominantly Zulu, or whatever, there should be a translator at hand for those patients who can’t speak English. Then we can get histories. I think that would benefit the practice and help patients so that we know what’s going on.’ (code 3:1)

‘It would help, but how many translators would there have to be? What language would they be able to help us with?’ (code 1:3)

One participant recounted an event where an interpreter assisted via a conference call and suggested teleconferencing as a way of eliminating the need for an in-house interpreter:

‘The one interpreter, they didn’t even come in, but they spoke to the patient over the phone and translated like that. They had like a conference call, doctor – patients, you know. So it wasn’t like the interpreter, they don’t always have to be there.’ (code 3:3)

### Mobile translation technology

Participants felt that technology could help overcome language barriers when an interpreter was not available. Mobile translation applications were recommended as smartphones allow for some applications to be downloaded at no cost. The possibility of a mobile application, specifically developed for the medical field, was raised in order to convey medical terminology accurately:

‘… [*P*]erhaps like Google Translate. Or something that is more modern and made for the medical field.’ (code 4:4)

The use of a mobile translation application that plays recordings was also suggested to help with the correct pronunciation of words:

‘Put it on an app as well, because it’s no use to me reading it and I don’t know how to pronounce it as well.’ (code 3:3)

### Employ a diverse workforce

Diversity can be challenging, but it also holds advantages. Employment of a diverse workforce was recommended by participants as a solution to overcome language barriers because it would increase the diversity in languages spoken amongst radiographers, whilst also reducing the cost of employing a dedicated translator. Participants further recommended the employment of radiographers who are proficient in the languages most commonly spoken in a specific area in a quest to address the language needs of local patients:

‘They could try and employ a person. Maybe a radiographer who speaks the language of that area. Like make a more diverse group.’ (code 3:1)

‘If they know that they have radiographers who don’t speak that language and if they are in that area where Zulu is common then they should out of their own employ someone who can speak that language.’ (code 3:1)

Participants additionally felt that a diverse and multilingual workforce makes it easier to facilitate informal communication with patients from different cultures. Having staff members who speak the language at hand ease communication, especially in stressful or emergency situations where a professional interpreter cannot be sourced:

‘I had found having multi-cultural radiographers now has made it much easier in the new South Africa. Because then you can go and ask one of your fellow radiographers if they do speak or understand that language.’ (code 4:5)

‘I think it comes definitely from the radiology side, because the thing is, a patient in a car accident doesn’t know they are going to be in an accident today. And if they are driving by themselves and they are brought to hospital, then they can’t communicate. So, I think from the department side, if you know that the common ground or the common type of people that you are getting in, at least just have one staff member that can communicate.’ (code 3:1)

The above quotes demonstrate the value of including a diverse workforce in overcoming language barriers.

### Tertiary framework

Even though SA is a lingually diverse country, English is the main language of instruction at tertiary level in a bid to accommodate the majority of students (Dube 2017:14). To broaden the language spectrum and minimise the language gap, participants recommended that language courses should be included in the curriculum:

‘Maybe they can bring it into the basic radiography training. To just have a little bit, like a module like that. Learning a few other languages to communicate.’ (code 3:5)

However, some participants affirmed that although they had the option to attend a Zulu course at university, it was later cancelled because of lack of interest. This led to a very interesting discussion about students’ perceptions regarding what they need to learn and what is important to know once you enter the workforce. It became clear that participants blamed their reluctance to participate in a language course on their ignorance of the importance of communication in different languages in an everyday radiography setting. However, once the need was identified, their perspective changed:

‘… [*I*]f it’s forced on you, you get the same thing “I’m going to learn the basics and I’m going to pass it, it’s another subject, whatever.” Once you are in the field and in the environment, you understand what your needs are. And then you feel like, or personally I feel like “now I would like to learn something to help my patients.” But if you forced it on me earlier and say you have to learn this because you are going to need it, I would’ve been more resistant. I would have done just the basics … But I think you don’t understand that when it is being forced on you at university.’ (code 4:1)

‘Because at varsity you don’t learn it because you don’t think you need it. Once you have been in the field for a year or two you realise that I am actually going to need this and then I think people are more responsive to it.’ (code 4:1)

Language courses at tertiary level can therefore be helpful in bridging the communication gap whilst allowing students to acquire a valuable skill. Nonetheless, the need to learn a language should be realised by the students whilst a variety of languages should be available to accommodate the multilingual nature of the student community.

## Discussion

Communication plays a vital role in delivering accurate and high-quality services. However, it is often taken for granted, its complexities and the innuendos are poorly understood and it receives inadequate attention when it comes to multicultural, multilingual communication (Baddley 2018:144). Effective communication is needed to facilitate cooperation and understanding between patients and radiographers. It furthermore empowers the patient with the understanding of what is expected and helps the radiology team to make an accurate diagnosis. The information that emerged from this study indicated that patient–radiographer communication is ineffective when it comes to cross-cultural communication whilst language barriers are everyday occurrences. The findings of this study can hence be extended to the rest of South Africa, as the country is a melting pot of diversity. As interaction between patients from different cultural and linguistic backgrounds occurs daily, the recommendations set forth from this study can be applied in other radiography settings in SA.

Data gathered from this study outline the experiences of radiographers communicating in a multicultural, multilingual healthcare setting. Subsequently, recommendations flowing from the data were tested against available literature.

### Workshops and short courses

Ideally these courses should be designed to empower radiographers with the cultural and linguistic skills needed to communicate with patients in a radiography setting. The notion of learning a new language for health workers to communicate better with their patients was echoed by Van den Berg (2016:230). Being able to converse with patients in their own language helps to build respect, trust and rapport between the patient and the caregiver (Van den Berg 2016:230). Maphosho (2018:146) further argued that when health workers can converse in the most common language spoken in a particular region, the need for an interpreter and risk of not knowing if your message is accurately interpreted and relayed to the patient is minimised. Having this skill, thus, empowers radiographers and patients alike (Maphosho 2018:146) as patients will be able to explain their symptoms in their home language.

Schlemmer and Marsh (2006:1087) suggested that courses in communication skills will empower staff to serve patients more effectively and efficiently whilst Juckett and Unger (2014:477) recommended that multilingual staff should additionally receive training in interpretation techniques as fluency in a language does not necessarily make them effective interpreters.

This could be achieved through continuous professional development (CPD) accredited workshops, because learning a new language could be regarded as professional development.

*Continuing education* units (CEUs) could act as an incentive for radiographers to attend these sessions (Murphy, Cross & McGuire 2006:369). Aboul-Enein and Ahmed (2006:169) encouraged healthcare workers to attend workshops and courses that focus on language and cultural awareness as a means to overcome communication barriers. Whilst patient needs are identified through verbal and non-verbal communication (Brooks, Manias & Bloomer 2018:3), culturally sensitive communication is crucial to ensure patient satisfaction (Khalid 2015:421).

### Posters and written documents

Participants strongly recommended the use of visual aids to help overcome language and communication barriers. Likewise, Tack et al. (2014:528) encouraged the use of imagery to enhance communication between patients and healthcare workers when adequate and effective communication cannot be achieved otherwise.

Although posters, pictograms and multilingual guidebooks can be useful tools in order to facilitate communication, the main limitation of this method is that communication becomes one-sided. Whilst the radiographer is able to give instructions, it does not allow for meaningful interaction when the patient attempts to communicate back (Finke, Light & Kitko 2008:2114).

Written communication strategies, however, offer considerable advantages because they can help to overcome the difficulty in describing symptoms or actions when there are cultural or language barriers (James, Cardew & Warren-Forward 2013:295). It additionally offers a cost-effective solution to overcome communication barriers.

Subsequently, pictures depicting certain actions such as falling, coughing or vomiting can be useful during the history taking process, whilst pictures of the body can be helpful for anatomical reference when the source of pain needs to be located (Finke et al. 2008:2104).

This method also benefits illiterate patients because pictures are self-explanatory. This concept has been employed successfully for many years with regard to radiation protection of a foetus in pregnant patients (James et al. 2013:269).

### Professional interpreter

Although radiographers should strive to preserve the confidentiality of their patients, they should also be able to identify when the help of a professional medical interpreter is required (Baylor et al. 2019:156). The purpose of professional interpreters in healthcare is to accurately recreate the meaning of what is being said between two parties who do not share a common language without omission, addition or change (Crezee & Roat 2019:2). Being trained to facilitate positive and meaningful interactions between two parties (Wang 2016:246), professional interpreters seem to provide the best solution to overcome language barriers.

However, in South Africa, the implementation of such a strategy may not be best suited because of the high costs involved and the number of official and foreign languages, which will have to be accommodated (Van den Berg 2016:230). Trained interpreters are also not readily available in the public health sector. Furthermore, the dialect of a specific region may also prohibit the interpreter to understand the patients completely (Thompson 2017).

Interpretation via a conference call could be explored as an option when a department or hospital could not afford an in-house interpreter (Van den Berg 2016:230). Subsequently, offsite interpreters are able to serve multiple facilities from a central location. The establishment of such a call centre would furthermore accommodate the diversity of local and international patients who use medical services in SA.

Whilst the use of a professional interpreter facilitates two-way communication between a radiographer and the patient, ethical considerations such as patient confidentiality and privacy should be taken into account when sensitive information is shared or when informed consent needs to be obtained (Carnevale et al. 2009:821).

### Mobile translation technology

The widespread use of telemedicine and telehealth has led to an increased acceptance of technology in healthcare. The use of technology has the potential to improve everyday clinical communication between patients and staff, reducing misunderstandings and improving patient care (Panayiotou et al. 2019:2).

A mobile application capable of playing a recording of the translated statement will help with the pronunciation of words and make it easier for the patient to understand (Scott-Weekly, Watts & Zacharias 2015:669). However, Patil and Davies (2014:7395) concluded that African languages scored the lowest in translation accuracy, with only 45% of the translation being accurate, followed by Asian languages with a 46% accuracy. Unfortunately, word-for-word translations often result in considerable changes in the intended meaning of a sentence and, therefore, these applications should be used with caution, especially when consent for a procedure is required (Beh & Canty 2015:669; Scott-Weekly et al. 2015:669) concluded that although mobile translation applications are not accurate enough to replace professional interpreters, they might be useful if an interpreter is not available.

Whilst older patients who are unfamiliar with technology may have difficulty using the application, it is also not guaranteed that the target language is available (Juckett & Unger 2014:477). Currently, Google Translate is only able to provide translations for five out of the 11 official SA languages (Temidoya 2020). Advancements in artificial intelligence (AI) could possibly put an end to language barriers across the world altogether (Rands 2018). Translations with the use of AI are able to translate an entire sentence instantly and accurately with access to thousands of languages. Additional factors to take into consideration are data security and confidentiality because some applications keep a record of the conversation. Thus, disclosing the patient’s identity and discussing sensitive information should be avoided when using a mobile application (Panayiotou et al. 2019:9).

Taking all facts into consideration, translation technology currently does not seem to be a suitable substitute for professional interpreters, necessitating further research regarding its use in a medical setting (Juckett & Unger 2014:477; Panayiotou et al. 2019:9; Patil & Davies 2014:7395; Scott-Weekly et al. 2015:669). However, it can offer some advantages in overcoming language barriers when it is not logistically or financially feasible to access professional interpreters. There is, hence, a pressing need to develop tools that can facilitate communication in a safe and effective manner and translation technology can play an important role in this regard (Beh & Canty 2015:669).

### Employ a diverse workforce

Language barriers arise throughout the healthcare system because of the high global migration rate and the diversity of the South African population (Census 2011). In order to respond to the demographic shift in the patient population, healthcare organisations are encouraged to employ a workforce that reflects the diversity of the patient base with respect to culture and language (Weech-Maldonado et al. 2002:112).

Mitchell and Lassiter (2006:2094) correctly argued that the absence of diversity in the workplace increases the potential for linguistic and cultural barriers, stifling access to high-quality healthcare for minority groups. Employing a culturally diverse workforce in a radiology department can, thus, help to overcome cultural and linguistic barriers between patients and radiographers.

It is common practice for radiographers to use colleagues as interpreters in order to overcome language barriers. Having a staff member at hand who can communicate with a patient in their preferred language helps avoid confusion and misunderstanding. Although Czapka, Gerwing and Sagbakken (2019:761) advised against this practice because of the breach in patient confidentiality, it is still seen as a viable route for communication.

Furthermore, a diverse workforce can help to advance cultural competency amongst employees by allowing individuals from varied ethnic and racial backgrounds to interact with each other (Cohen, Gabriel & Terrel 2002:90). This will facilitate a shared cultural understanding, which will in turn have a beneficial effect on patient care (Cohen et al. 2002:100).

### Tertiary framework

‘Cultural responsiveness’ in healthcare aims to address factors that may contribute to racial or ethical disparities. It describes the ability to provide care to patients with diverse values, beliefs and behaviours, including tailoring service delivery to meet the patient’s social, cultural and linguistic needs. The ultimate goal of a radiographer should, therefore, be to deliver the highest quality of care to every patient, regardless of race, ethnicity, cultural background or language proficiency (Betancourt et al. 2005:501).

To this effect, intercultural communication competence plays a crucial role in preparing a student to be an effective communicator. However, currently, there is limited literature on how to prepare health professionals to provide culturally competent care in the SA context (Matthews & Van Wyk 2018:113). It is thus highly recommended that the concepts of cultural responsiveness and cultural sensitivity should be promoted and introduced in the curricula of healthcare professionals to improve service delivery and provide the necessary support to linguistically and culturally diverse groups (Matthews & Van Wyk 2018:118). By understanding the relationship between cultural beliefs and behaviour and developing skills to improve quality of care to diverse populations, radiographers will be empowered to provide an efficient and effective service to all patients (Betancourt et al. 2005:501).

Understanding the non-visible aspects of culture, such as health beliefs, communication style and religion, will additionally assist radiographers in becoming more attuned to the culturally based health expectations held by patients with different cultural backgrounds (Kreuter & McClure 2004:440).

In addition, as recommended by the participants, language courses incorporated in the curriculum may give students a head start in facilitating multilingual communication between patients and themselves. Given the scale of migration and globalisation, the ability to communicate in more than one language has become even more pressing (Betancourt et al. 2005:502).

However, the different needs of a diverse student body imply that a number of language courses might have to be implemented, which may prove challenging. Furthermore, in order for a language course to be successful, students need to realise the importance of effective communication with patients from different backgrounds. Garmashova (1997:131) concluded that South African university students regard learning a new language more as a hobby than a professional requirement. Subsequently, existing programmes should be revised and new study materials should be compiled to be compatible with and relevant to the profession or the radiography context in particular (Betancourt et al. 2005:504).

### Limitations of the study

The sample used in this study was not sufficiently representative of the radiography population of Gauteng province in terms of race, gender and language spoken. It could be argued that the views presented here are mainly that of white, English-speaking, female radiographers.

## Conclusion

Radiology departments should consider the consequences and the resulting cost associated with language barriers and the toll it can take on providing effective and efficient service. Employing a designated professional interpreter may not be financially possible, but there are alternative options for health facilities in order to decrease the financial burden of language barriers. However, employing a diverse workforce or utilising posters in radiography departments can offer a cost-effective alternative and improve the quality of care provided. Furthermore, workshops or short courses and the inclusion of ‘cultural responsiveness’ in the tertiary framework will empower radiographers with the tools to approach patients from different cultural backgrounds in order to serve them to the best of their ability. The use of mobile translation technology as a way to overcome communication barriers should be explored further.

Given the fact that the South African Constitution’s Bill of Rights stipulates that the healthcare needs and cultures of all citizens must be embraced, culturally sensitive communication is one of the most important tools to ensure high-quality patient care and satisfaction. Although a variety of communication strategies are available to overcome communication barriers in a multicultural setting, the most appropriate combination should be explored for individual radiology practices in order to serve their respective diverse patient base.
